# Report of a novel missense mutation in the *MECP2* gene in a middle‐aged man with intellectual disability syndrome

**DOI:** 10.1002/ccr3.4602

**Published:** 2021-08-21

**Authors:** Maria Arvio, Maria Haanpää, Pia Pohjola, Jaana Lähdetie

**Affiliations:** ^1^ Neurology Päijät‐Häme Joint Municipal Authority Lahti Finland; ^2^ PEDEGO Oulu University Hospital Oulu Finland; ^3^ Southwest Special Care Municipal Authority Paimio Finland; ^4^ Department of Child Neurology Turku University Hospital Turku Finland; ^5^ Department of Genomics Turku University Hospital Turku Finland; ^6^ Turku University Turku Finland

**Keywords:** intellectual disability, MECP2, movement disorder, progressive disorder

## Abstract

Exome sequencing revealed the cause of our 35‐year‐old male patient's progressive and severe intellectual and motor disability, namely a previously undescribed missense mutation of MECP2.

## Graphical abstract

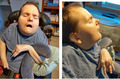

## 1 INTRODUCTION

We describe the natural history of a 35‐year‐old man with progressive intellectual and motor disability, short stature, epilepsy, sleep, eating, and movement disorders. Exome sequencing revealed the genetic cause of his symptoms. A likely pathogenic variant in the MECP2 gene was most probably the cause for his progressive disorder.

The methyl‐CpG‐binding protein 2 (*MECP2*) gene is mainly known by its variants causing Rett syndrome (MIM 312750) in females. This gene is located in the X chromosome, and there are several phenotypic expressions of the mutations thought to follow X‐linked dominant inheritance with a high rate of early loss in hemizygous male pregnancies. The typical Rett syndrome consists of initially normal development followed by autistic regression and characterized by loss of spoken language and hand use with the development of distinctive hand stereotypies. Other characteristic features include seizures, motor impairment, and intellectual disability (ID).^1^

Male patients with *MECP2* pathogenic variants show higher phenotypic variation than previously expected.^2^ Male patients can be classified into three categories,the first one being *MECP2*‐related severe neonatal encephalopathy,^3^ the second PPM‐X syndrome/pyramidal signs, psychosis/parkinsonism, and macro‐orchidism syndrome,^4^, ^5^, ^6^, ^7^, ^8^ and the third syndromic/nonsyndromic ID syndromes^6^, ^9^, ^10^, ^11^, ^12^, ^13^ (Table 1).

**TABLE 1:** Features in the three phenotypes of *MECP2* disorders in males compared with the features of our patient

*MECP2*‐related severe neonatal encephalopathy	Present at least in (%)	Our patient
Normal birth parameters	71	+
Head growth deceleration/microcephaly	94	−
Hypotonia and/or feeding difficulties in infancy	82.4	+
Hypertonia of extremities	52.9	+
Movement disorder, for example, myoclonus, tremors, and dystonia	58.8	+
Mild cerebral atrophy	18	−
Polymicrogyria	5.9	−
Poor head control	35	+
Seizures	58.8	+
Severe developmental delay	82.4	+
Irregular breathing/sleep apnea	47.1	+
Gastroesophageal reflux	35.3	+
EEG abnormality	88.2	+

Pyramidal signs, parkinsonism, and macroorchidism (PPM‐X syndrome)	Present at least in (%)	Our patient
Psychosis	67.6	−
Pyramidal signs	46	+
Macroorchidism	19	−
Intellectual disability	50	+
Parkinsonism	2.7	+
Progressive spasticity	67.6	+
Delayed development	54	+
Speech difficulties	50	+
Seizures	2.7	+
Bilateral juvenile cataract	2.7	−
Scoliosis or kyphosis	10.8	+
Large ears	8.1	−
Movement disorder	32.4	+
Apraxia	2.7	+
Seizures	8.1	+
Dysmorphic features	5.4	−

Syndromic/nonsyndromic intellectual disability	Present at least in (%)	Our patient
Severe intellectual disability	90	+
Gait abnormalities	57	+
Facial dysmorphism	10	−
Behavioral problems	40	−
Autistic‐like behavior	3	−
Seizures	20	+
Poor/absent language skills	47	+
Hypotonia	23	−
Microcephaly	13	−
History of regression	17	+
Spasticity	33	+
Sleep disturbances	13	+

We describe a detailed history of a 35‐year‐old man. After several and expensive investigations, many of them done under general anesthesia, clinical exome sequencing revealed the genetic cause of his multiple disabilities and progressive disorder.

Clinical data and images are published with the consent of the patient's mother.

## 2 CASE DESCRIPTION

The patient is the only child of a healthy non‐consanguineous couple. The mother has two daughters with another man; one half‐sister has a mild ID of unknown cause. The patient was born after an uncomplicated pregnancy by cesarean section. His birth measurements were normal, weight being 4230 g (+1.3 SD), height 51 cm (+0.3 SD), and head circumference 37 cm (+0.6 SD). At the age of 5 months, a slight systolic murmur was recorded.

By the age of 1 year 8 months, the patient could walk clumsily and he had ataxia. Tendon reflexes were brisk. He did not speak any words. From the age of 2 years, he suffered from generalized tonic‐clonic seizures. The electroencephalogram showed centro‐parietal spikes as well as and spike‐wave bursts suggesting focal epilepsy. After the initiation of carbamazepine, the seizures ceased. The etiology of his overall delayed development, ataxia, and epilepsy remained unknown in spite of a large amount of examinations. Findings in brain magnetic resonance image (MRI), single‐photon emission computerized tomography, electroretinography, and visual evoked potential study were normal. Neither did karyotype, metabolic screening, rectal biopsy, fragile X studies nor muscle enzyme tests (among other studies) reveal any abnormal results. The only abnormal finding was bilaterally delayed somato‐sensory evoked potentials.

**FIGURE 1:**
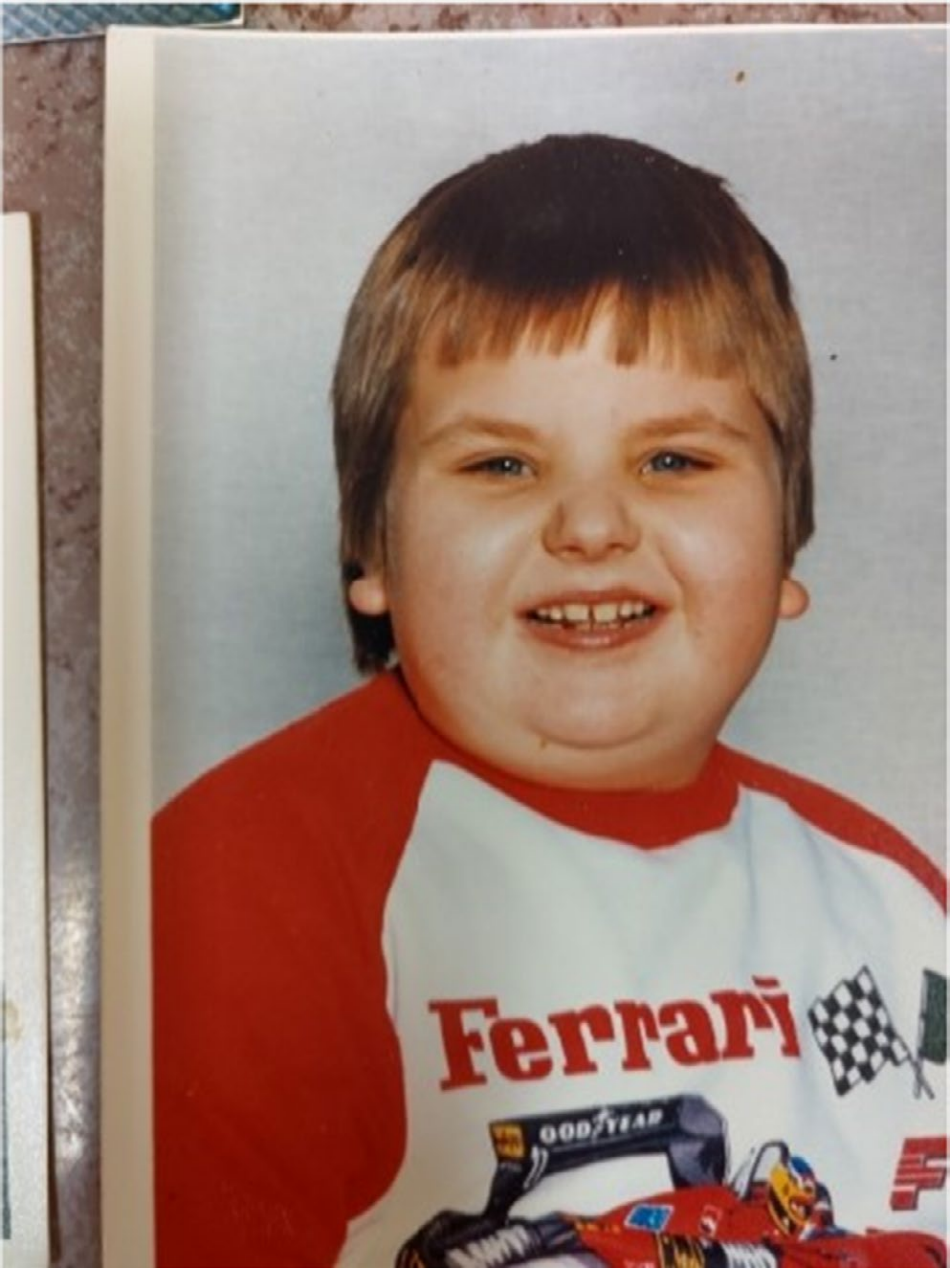
Patient at the age of 3 years

At the age of 3 years, he could run and climb stairs. He has no dysmorphic features except a peculiar shape of earlobes (Figure 1).

At the age of 6 years, obesity (weight +110%), tendency to overeat, vomiting, sleep apnea, and pavor nocturnus aroused a suspicion of Prader‐Willi syndrome but genetic testing of this syndrome was normal.

At the age of 9 years, he could speak single words and used some sign language in communication.

**FIGURE 2:**
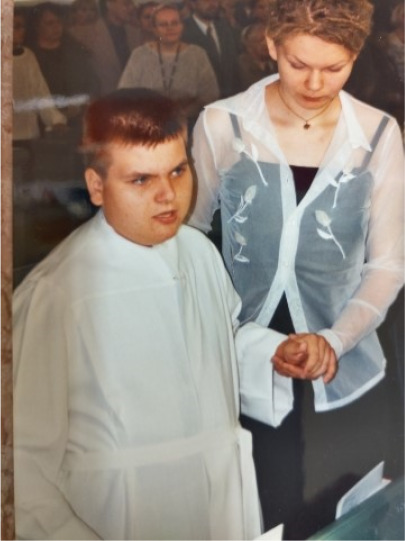
Patients at the age of 15 years

At the age of 15 years, he acquired the skills of independent in toileting and dressing (Figure 2). He biked with a three‐wheeled bicycle, but climbing upstairs had become too challenging, although according to motor function tests, his muscle strength was normal. He had slight kyphosis. Brain MRI was repeated and the brain was normal, but an abnormally thick calvarium was observed.

From the age of 17 years, he experienced a severe decline in motor skills. Ataxia became evident again and within 5 years he lost all motor skills and became spastic resulting in joint contractures. He underwent an orthopedic procedure of the knees and an operation of sinus pilonidalis. Epileptic seizures relapsed, and as a new sign, he developed a movement disorder manifesting as 10 min lasting stiffness attacks, resembling those observed in Parkinson's disease. In spite of rapid clinical deterioration, arising the suspicion of a neurodegenerative disease, there was no change in the third, updated brain MRI, compared to earlier ones.

**FIGURE 3:**
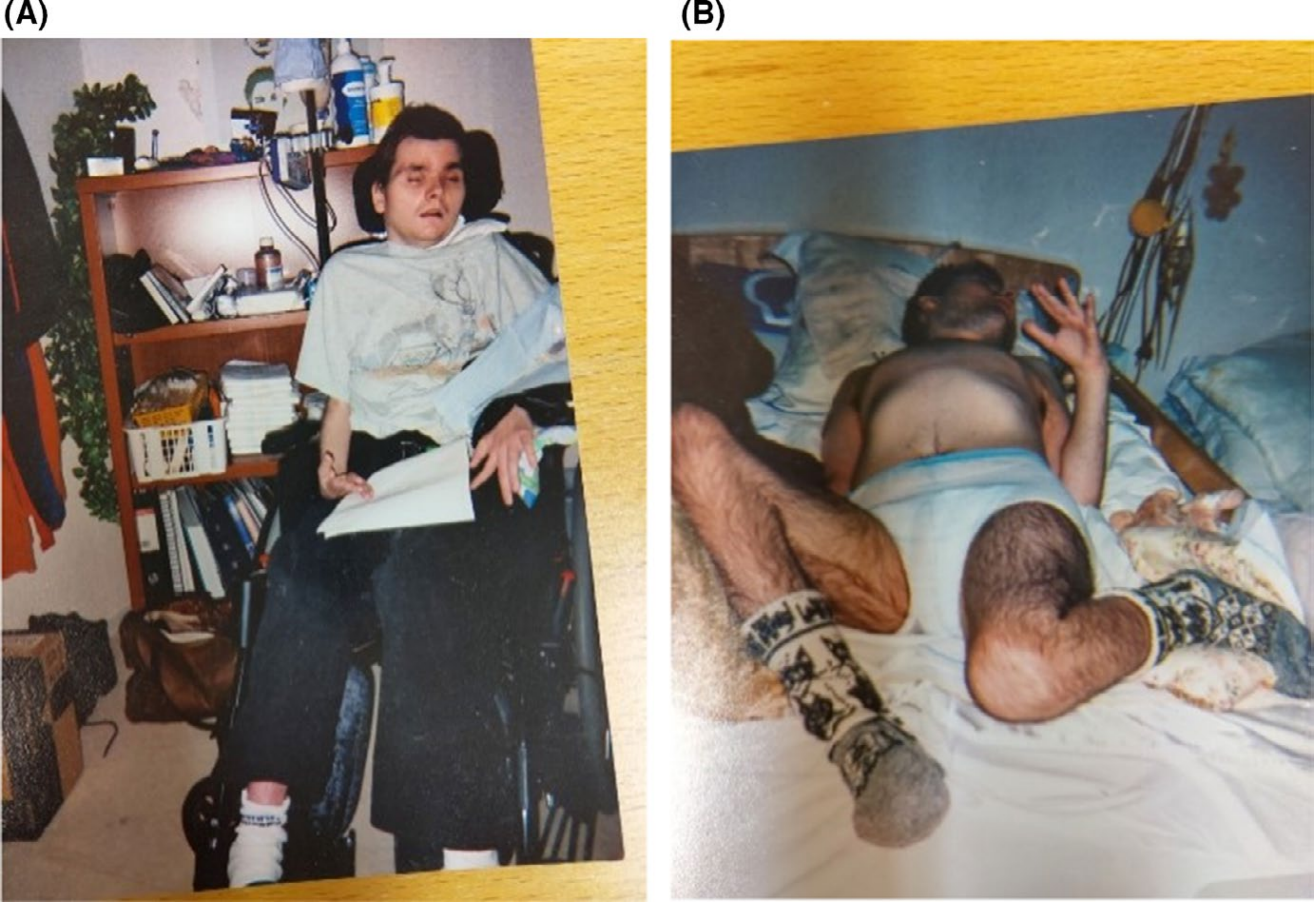
(A, B) Patient at the age of 24 years

At the age of 24 years, he stopped swallowing and lost weight (Figure 3A,B). Because of underweight, he underwent gastrostomy and tube nutrition was started. It turned out to be a pivotal procedure since soon after this operation the patient's condition ameliorated and he started to eat normally.

**FIGURE 4:**
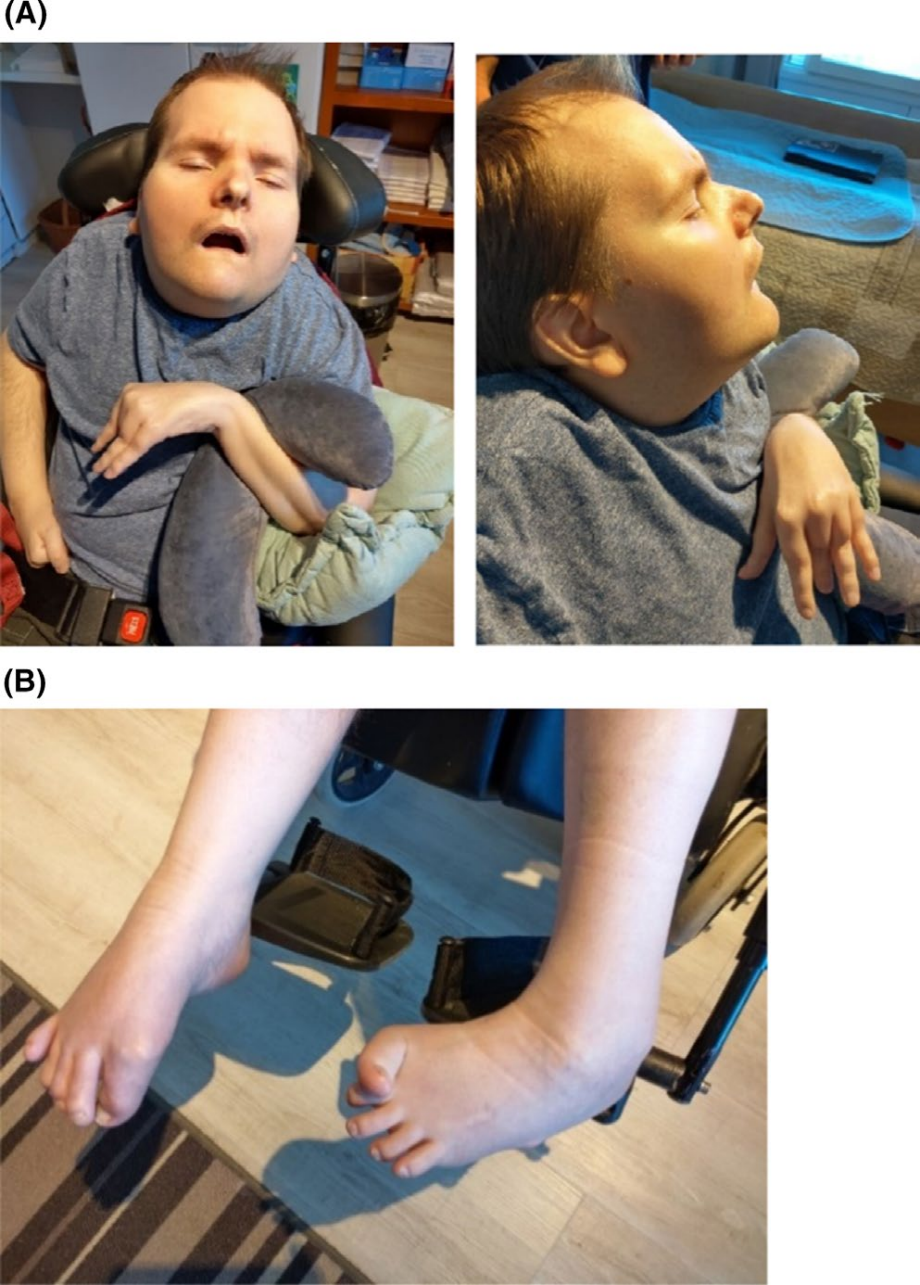
(A, B) Patient at the age of 35 years

His condition has now been stable for over 10 years. At present, he communicates with gaze and head nodding and laughs to jokes. His legs do not bear and when sitting he needs full support. He has several joint contractures, slight scoliosis, and club‐foot (Figure 4A,B). He is seizure free, and neither are there signs of Parkinsonism. Tendon reflexes are brisk. He has short stature: his height is 150 cm (−5 SD), weight 80 kg, and head circumference 59 cm.

Etiological studies were updated. Brain MRI finding, repeated for the fourth time, was normal, as earlier. For genetic analyses, DNA was extracted from peripheral blood. Microarray did not show any clinically relevant copy number variants. A NGS‐based analysis was performed with Sophia Genetics custom clinical exome solution including 4430 disease‐related genes and Illumina sequencing. Over 98% of target regions was covered with 50×. Variant annotation and filtering were performed with Sophia DDM and visualized with IGV (Broad Institute). The analysis revealed a novel hemizygous likely pathogenic missense variant in the *MECP2* gene, c.500G>C, p.(Arg167Pro) which has not been described in databases (gnomAD, ClinVar, LOVD, HGMD Professional) or in the literature. The variant localizes in exon four, changes an evolutionally conserved amino acid from arginine to proline, and affects the important methyl‐CpG‐binding domain of the protein. In silico tools predict that the variant found in our patient is pathogenic. Another missense variant, in the same codon (c.499C>T), has been described earlier, altering arginine to tryptophan, and it is likely pathogenic in male patients.^14^, ^15^ Patients with this alteration have variable phenotypes.

We compared the clinical picture of our patient to the summary descriptions of *MECP2*‐related severe neonatal encephalopathy, of PPM‐X syndrome, and of *MECP2*‐related syndromic/nonsyndromic ID syndromes designed by Kaur and Christodoulou^16^ (Table 1). Our patient shows large overlapping with all three phenotypes.

The mother and the half‐sister with a mild ID have not given their consent to genetic testing.

## 3 DISCUSSION

Molecular‐genetic techniques have revealed the causes of disabilities of several patients during the recent years. This is of great importance because the knowledge helps in treatment, rehabilitation as well as in when considering the prognosis for the patient. Genetic causes may be hereditary and the knowledge enables genetic counseling to the families. Knowledge may also relief the parents from useless speculation in cases when the cause is unknown.

Our patient's symptoms include severe intellectual and motor disability, poor communication, short stature, sleep disorder, eating disorder, epilepsy, and movement disorder resembling Parkinsonism. According to a recent review, there are 183 X‐linked ID syndromes and 141 ID genes in the X chromosome.^2^ One gene may have more than one phenotypic manifestation depending on the mutation. This is the case with the *MECP2* gene in males.

DNA methylation is the major mechanism of modification of eukaryotic genomes and plays an essential role in mammalian development. MECP2 belongs to a family of nuclear proteins related by the presence in each of a methyl‐CpG‐binding domain (MBD). MECP2 can repress transcription from methylated gene promoters. It is required for the maturation of neurons and is developmentally regulated. Since MECP2 is X‐linked, it is a subject to X inactivation. MECP2 is essential for embryonic development. *MECP2* gene mutations are responsible for most of the cases of Rett syndrome which is only observed in females. In male embryos, these mutations were considered to be lethal. However, there have been clear reports that some pathogenic variants in *MECP2* are compatible with life in men and there are at least three different phenotypes.^3^, ^4^, ^6^, ^7^ For example, the recurrent mutation p.(Ala140Val) is well known, affecting the methyl‐CpG‐binding domain^4^, ^17^ yielding a slightly less severe phenotype and carrier mothers have been unaffected (Cohen, 2002).

When evaluating a male patient with an ID and somatic, neurologic, or behavior manifestations such as in the present case, a large number of syndromes need to be considered for differential diagnoses. Family history of our patient suggested X‐chromosomal inheritance due to the mildly affected half‐sister. Physical examination and the natural history suggested a progressive, neurodegenerative disease, explaining why MRI was repeated so many times and metabolic diseases searched with such a wide variety of laboratory test without success. Fortunately, genetic techniques are extremely helpful today. Microarray copy number variation is detected in 15% and pathogenic variants in whole‐exome sequencing (WES) in 40% of ID patients with unknown etiology.^18^ Due to its usefulness and high rate of diagnostic yield, WES has become the primary etiological investigation of syndromic and nonsyndromic ID.^18^, ^19^ It has been shown to save on average 3547 dollars in cases where genetic or metabolic etiology is suspected.^19^

In our case, clinical exome analysis finally gave the name for the syndrome of our patient. Many years, many laborious investigations, and a lot of money had already been used to find an etiological diagnosis for him. Since the variant of the *MECP2* gene, that was observed in his DNA, is previously undescribed but changes an evolutionally conserved amino acid in a functionally important domain (methyl‐CpG‐binding domain) of the protein, and since the clinical manifestations show much overlap with all three the previously reported male MECP2‐induced neurodegenerative conditions, we are convinced that this variant c.500G>C, p.(Arg167Pro) is the causative genetic alteration in our patient. Confirmation of inheritance by analyzing the mother's and half‐sister's DNA would have been valuable.

In conclusion, we present the natural history of the oldest male patient described in the medical literature. Our 35‐year‐old patient has a previously undescribed *MECP2* likely pathogenic variant. There are now numerous male patients reported to have *MECP2* mutation‐caused disorders and thus our paper further confirms this MECP2‐related syndrome in males and extends the phenotypic spectrum. The clinical spectrum of neurodevelopmental symptoms and other syndromic features may help clinicians to identify other patients with similar ID syndrome.

## CONFLICT OF INTEREST

None declared.

## AUTHOR CONTRIBUTIONS

The corresponding author MA has followed the clinical course of the patient. MH and PP performed the exome sequencing. JL contributed with expert knowledge in clinical genetics and neurology.

## ETHICAL APPROVAL

Our manuscript is not published elsewhere. All co‐authors meet the criteria for authorship.

## Data Availability

The data that support the findings of this study are available from the corresponding author upon reasonable request.

## References

[1] Gold WA, Krishnarajy R, Ellaway C, Christodoulou J. Rett syndrome: a genetic update and clinical review focusing on comorbidities. ACS Chem Neurosci. 2018;21:167‐176. 10.1021/acschemneuro.7b00346 29185709

[2] Neri G, Schwartz CE, Lubs HA, Stevenson RE. X‐linked intellectual disability update 2017. Am J Med Genet A. 2018;176(6):1375‐1388. 10.1002/ajmg.a.38710 29696803 PMC6049830

[3] Schüle B, Armstrong DD, Vogel H, Oviedo A, Francke U. Severe congenital encephalopathy caused by MECP2 null mutations in males: central hypoxia and reduced neuronal dendritic structure. Clin Genet. 2008;74(2):116‐126. 10.1111/j.1399-0004.2008.01005.x 18477000

[4] Klauck SM, Lindsay S, Beyer KS, Splitt M, Burn J, Poustka A. A mutation hot spot for nonspecific X‐linked mental retardation in the MECP2 gene causes the PPM‐X syndrome. Am J Hum Genet. 2002;70(4):1034‐1037. 10.1086/339553.15 11885030 PMC379098

[5] Moog U, Smeets EE, van Roozendaal KE, et al. Neurodevelopmental disorders in males related to the gene causing Rett syndrome in females (MECP2). Eur J Paediatr Neurol. 2003;7:5‐12. 10.1016/s1090-3798(02)00134-4 12615169

[6] Orrico A, Lam C, Galli L, et al. MECP2 mutation in male patients with non‐specific X‐linked mental retardation. FEBS Lett. 2000;22(481):285‐288. 10.1016/s0014-5793(00)01994-3 11007980

[7] Psoni S, Sofocleous C, Traeger‐Synodinos J, Kitsiou‐Tzeli S, Kanavakis E, Fryssira‐Kanioura H. Phenotypic and genotypic variability in four males with MECP2 gene sequence aberrations including a novel deletion. Pediatr Res. 2010;67(5):551‐556. 10.1203/PDR.0b013e3181d4ecf7 20098342

[8] Winnepenninckx B, Errijgers V, Hayez‐Delatte F, Reyniers E, Frank Kooy R. Identification of a family with nonspecific mental retardation (MRX79) with the A140V mutation in the MECP2 gene: is there a need for routine screening? Hum Mutat. 2002;20(4):249‐252. 10.1002/humu.10130 12325019

[9] Gomot M, Gendrot C, Verloes A, et al. MECP2 gene mutations in non‐syndromic X‐linked mental retardation: phenotype‐genotype correlation. Am J Med Genet A. 2003;123A(2):129‐139. 10.1002/ajmg.a.20247 14598336

[10] Meins M, Lehmann J, Gerresheim F, et al. Submicroscopic duplication in Xq28 causes increased expression of the MECP2 gene in a boy with severe mental retardation and features of Rett syndrome. J Med Genet. 2005;42(2):e12. 10.1136/jmg.2004.023804 15689435 PMC1735993

[11] Meloni I, Bruttini M, Longo I, et al. A mutation in the rett syndrome gene, MECP2, causes X‐linked mental retardation and progressive spasticity in males. Am J Hum Genet. 2000;67(4):982‐985. 10.1086/303078 10986043 PMC1287900

[12] Ramocki MB, Peters SU, Tavyev YJ, et al. Autism and other neuropsychiatric symptoms are prevalent in individuals with MeCP2 duplication syndrome. Ann Neurol. 2009;66(6):771‐782. 10.1002/ana.21715 20035514 PMC2801873

[13] Van Esch H, Bauters M, Ignatius J, et al. Duplication of the MECP2 region is a frequent cause of severe mental retardation and progressive neurological symptoms in males. Am J Med Genet Part A. 2005;77(3):442‐453. 10.1086/444549 PMC1226209 16080119

[14] Neul JL, Benke TA, Marsh ED, et al. The array of clinical phenotypes of males with mutations in Methyl‐CpG binding protein 2. Am J Med Genet B Neuropsychiatr Genet. 2019;180:55‐67. 10.1002/ajmg.b.32707 30536762 PMC6488031

[15] Villard LJ. MECP2 mutations in males. Med Genet. 2007;44(7):417‐423. 10.1136/jmg.2007.049452 PMC2597995 17351020

[16] Kaur S, Christodoulou J. *MECP2* disorders. In: Adam MP, Ardinger HH, Pagon RA, et al., GeneReviews® [Internet]. University of Washington, Seattle; 1993‐2021. Bookshelf URL: https://www.ncbi.nlm.nih.gov/books/ NLM Citation. 2001 Oct 3 [Updated 2019 Sep 19]. 20301670

[17] Cohen D, Lazar G, Couvert P, et al. MECP2 mutation in a boy with language disorder and schizophrenia. Am J Psychiatry. 2002;159(1):148‐149. 10.1176/appi.ajp.159.1.148-a 11772708

[18] Bass N, Skuse D. Genetic testing in children and adolescents with intellectual disability. Curr Opin Psychiatry. 2018;31(6):490‐495. 10.1097/YCO.0000000000000456 30138136

[19] Monroe GR, Frederix GW, Savelberg SM, et al. Effectiveness of whole‐exome sequencing and costs of the traditional diagnostic trajectory in children with intellectual disability. Genet Med. 2016;18(9):949‐956. 10.1038/gim.2015.200 26845106

